# Memoir of the Late James Hope

**Published:** 1842-10

**Authors:** 


ART. VII.—*Memoir of the late James Hope*, M.D., *Physician to St. George's Hospital, &c. &c.* By Mrs. HOPE. Edited by KLEIN GRANT, M.D. &c. &c.—*London*, 1842. 8vo, pp. 358.

WE have already given, in our obituary, so full a biographical notice of Dr. Hope, that it is unnecessary for us to dwell on the events of his life as recorded in the volume before us, nor is it indeed within our province to enter minutely into the details of personal history, however worthy its object.

This memoir is one of great interest to the religious, the general, and the medical reader. As a religious production it would be wholly out of place for us to comment upon it further than to express our respect for the sentiments it contains, and our wish that it may be instrumental in advancing the cause of practical Christianity. In a professional and general light we would chiefly draw attention to it, as an illustration of the power of perseverance and concentration of the mind on a particular object; for to these it appears to us that the great and early success of Dr. Hope in his profession was mainly attributable. At home or abroad, in the cheerfulness of success or the gloom of disappointment, he kept in constant view the object of a laudable ambition—the attaining a high rank in his profession; and he continued, from an early period of his studies, to accumulate observations on a very difficult and important subject, till he produced the best work upon it that has yet appeared in our language. His discharge of all public duties evinced the same undeviating perseverance, and a conscientious desire of fulfilling to the utmost the trust reposed in him.

While his example in these respects may be held up as worthy of all imitation, his premature death may serve as a warning against excessive exertion and overstraining of the mental and bodily powers—a warning not to be neglected in an age when the difficulty of rising in the learned professions is so great that many men of high promise fall victims in early life to excess of labour. Dr. Hope's exertions at St. George's Hospital were truly immense, and the able and conscientious manner in which he fulfilled his functions demands for him, in full measure, the respect of his profession and the gratitude of the public, while it is impossible to repress a feeling of deep regret that his labours in such a field of usefulness should have been the principal exciting cause of the disease which proved fatal to him.

The memoir is extremely creditable to the talents, good sense, and good taste of its author, Mrs. Hope; and the editor, Dr. Grant, seems to have discharged his share of the task with much judgment and with a degree of prudence and reserve which editors of others' writings are not always disposed to show. In conclusion, we warmly recommend this work not only to our own readers but to the reading public generally. It will be found highly interesting in the perusal, and will leave no feeling of disappointment in the mind, having reference to the author, the editor, or the lamented subject of it.

